# Modulating human memory for complex scenes with artificially generated images

**DOI:** 10.1038/s41598-022-05623-y

**Published:** 2022-01-28

**Authors:** Cameron Kyle-Davidson, Adrian G. Bors, Karla K. Evans

**Affiliations:** 1 Department of Computer Science, University of York, York, YO10 5GH UK; 2 Department of Psychology, University of York, York, YO10 5DD UK

**Keywords:** Computer science, Psychology

## Abstract

Visual memory schemas (VMS) are two-dimensional memorability maps that capture the most memorable regions of a given scene, predicting with a high degree of consistency human observer’s memory for the same images. These maps are hypothesized to correlate with a mental framework of knowledge employed by humans to encode visual memories. In this study, we develop a generative model we term ‘MEMGAN’ constrained by extracted visual memory schemas that generates completely new complex scene images that vary based on their degree of predicted memorability. The generated populations of high and low memorability images are then evaluated for their memorability using a human observer experiment. We gather VMS maps for these generated images from participants in the memory experiment and compare these with the intended target VMS maps. Following the evaluation of observers’ memory performance through both VMS-defined memorability and hit rate, we find significantly superior memory performance by human observers for the highly memorable generated images compared to poorly memorable. Implementing and testing a construct from cognitive science allows us to generate images whose memorability we can manipulate at will, as well as providing a tool for further study of mental schemas in humans.

## Introduction

Cognitive science research of human visual episodic memory over the last few decades reveals both large storage capacity and a surprising ability to retain detail^1,2^. Recent work at the intersection between the fields of machine learning and cognitive psychology have exposed another property of visual memory for images: consistency between observers^3^. Showing a set of images to a human population sample, most members of that sample will remember roughly the same subset of images. This implies that to a certain extent, image memorability (i.e. how likely the average person is to remember a given image) is an implicit property of the image itself. Image memorability does not correlate strongly with simple image characteristics such as colour, intensity, the number of objects present in the scene^3^, or with attention, and is robust to overt cognitive influence^4^. Rather, high-level scene attributes help explain the memorability of images^5^, such as the content of the image (for example the presence of “a person”) or the dynamics occurring in the captured scene (“throwing a ball”). While memorability is affected very weakly by certain global features, such as average image hue and contrast, semantic context plays a stronger role, explored in^6^. These features are related to, but not completely explained by, objects present in the image^7^, and specifically, their location and size^8^. These findings have lead to attempts to predict image memorability using computational tools, which find the best predictor to be high-level semantics, such as the image scene category^5^. Later work established the influence of scene category and contextual distinctiveness on memorability^9^, and current state-of-the-art models employ automatic deep feature extraction via convolutional neural networks (CNN)^10,11^. The field of image memorability prediction has advanced to the point where CNN-based models can predict how likely an image is to be remembered with human-level consistency (Spearman rank correlation coefficient of $\rho = 0.67$)^10^.

Initially the majority of research studies framed the problem of image memorability prediction as regression to a one-dimensional score. Recent research results develop an understanding of memorability as a two-dimensional property that varies across an image, resulting in the extraction and analysis of cognitive relational patterns that capture the regions of scene images human observers deem memorable. These relational patterns, known as Visual Memory Schemas (VMS)^12^, capture the cognitive representations and structures that humans use to organise and encode a given image into memory, have high consistency between humans ($\rho = 0.70$), and a limited relation with one dimensional single score predictors for memorability. VMS internal consistency (measured via Pearsons 2D correlation) is higher than both VMS correlation with eye fixations ($P^{2D} = 0.50$) or saliency ($P^{2D} = 0.58$)^13^. Compared to image memorability prediction, fewer works tackle the task of modifying the memorability of images, or that of generating images that are intended to be less or more memorable. Modifying the memorability of face images was explored in^14^, where it was found that active appearance models^15^ could be employed to adjust various facial features associated with memorability. Deep generative models have also shown some success in modifying image memorability, from face generation^16^, to employing style transfer^17^, to transformer-based network capable of modifying the memorability of a seed image^10^.

Here, we develop a generative model we call ‘MEMGAN’, capable of synthesizing completely new photo-realistic scene images by using two-dimensional maps of memorability. These maps are based upon cognitive relational patterns, which reveal the mechanisms humans employ to encode scene images in memory. We validate this approach by performing a repeat-recognition human experiment, and find that our generated images significantly modulate the memory performance of human observers. When designing our approach, we set out to verify that visual memory schemas (VMS) capture information that is memorable in an image to a sufficient degree to constrain a generative model that can synthesise completely new scenes which in turn are able to modulate human memory. We start with the analyses of the per-category consistency and the per-category memorability signal (measured by D-Prime) for the VISCHEMA image datasets^18^, and explore the relationship between consistency and memorability in order to verify that VMS maps are suitable descriptors of memorability. We then consider two deep learning generative adversarial techniques for generating memorable images: based upon the Wasserstein loss metric^19^, and a progressively growing network. This approach allowed us to investigate what effect modifying the visual memory schemas the scenes were based on, has on the generated images. The generative neural network requires feedback on the memorability of its’ synthesised scenes during training time. The network generates hundreds of thousands of images during its training, and memorability feedback is necessary for every generated image. To deal with this constraint, we train a VMS prediction model based directly upon human data that can produce VMS feedback for arbitrary scene images. The predictor learns which features (from indoor scenes) make up a visual schema for our experimental kitchen scenes. It is this feedback that constrains the generative model. We evaluate our generated scenes via a human observer memory experiment, testing if our newly generated more memorable images are remembered better than the generated low memorability images. These findings allow us to acquire new insights into the efficacy of modulating the performance of human memory via images generated to activate specific visual memory schemas in human observers.

Developing the capability to generate memorable scene images without requiring an initial image seed has clear practical and theoretical applications. Such a technique could be applied to create highly effective memorable advertisements, improve educational tools, and there is also the potential for medical applications, such as tracking the decline in memory of patients with advancing cognitive deficits by providing a targeted baseline of memorability. A completely data driven approach such as this would provide significant advances to the methods used in cognitive science for the study of mental structures for the organisation of thought and behaviour employed by humans.

## Results

### VMS consistency and memorability

VMS maps capture spatial and relational components of episodic memory, and hence contain additional information compared to single-score based image memorability methods. In order to evaluate the validity of the data driven VMS maps as image memorability predictors we examine their image category consistency. We employ a method from signal detection theory to extract a global ‘memorability’ signal for each category for human observers and then evaluate the correlation between this global signal and VMS map consistency. The evaluation is performed on both the VISCHEMA 1 dataset^12^, which consists of 800 images and their corresponding 800 2D memorability maps, and the VISCHEMA 2 dataset^13^, an expansion to VISCHEMA 1 which consists of another 800 images and memorability maps.

The VISCHEMA 1 and 2 datasets contain a variety of images, grouped in the following categories: isolated, populated, public, entertainment, work/home, kitchen, living room, small and big. The consistency of the VMS maps, on a category-by-category bases for both VISCHEMA 1 and 2 is presented in Table 1. The consistency is calculated by taking 25 splits of the data (one split creating two VMS maps for each image, each built from an equal division of human annotation data) and correlating the resulting VMS maps against each other, using the Pearson’s Correlation Coefficient. For all image categories the correlation is positive, and in many cases, strongly positive as is the case for the “entertainment” category, composed of images of fairgrounds and playgrounds. Observers tend to agree with each other on which regions allowed them to remember the image in the categories that show strong consistency signal.

**Table 1:** Vischema 1 and Vischema 2 consistency, per category. Certain categories of images, such as kitchens or scenes involving public entertainment (playgrounds, theme parks) are more consistent than others, such as the isolated category. Higher consistency implies participants agreed on specific features that made the image memorable.

Category	Consistency	
	VISCHEMA 1	VISCHEMA 2
Isolated	0.556	0.447
Populated	0.624	0.562
Public Ent.	0.706	0.661
Work/home	0.674	0.57
Kitchen	0.628	0.479
Living room	0.568	0.446
Small	0.611	0.525
Big	0.637	0.595

**Figure 1:**
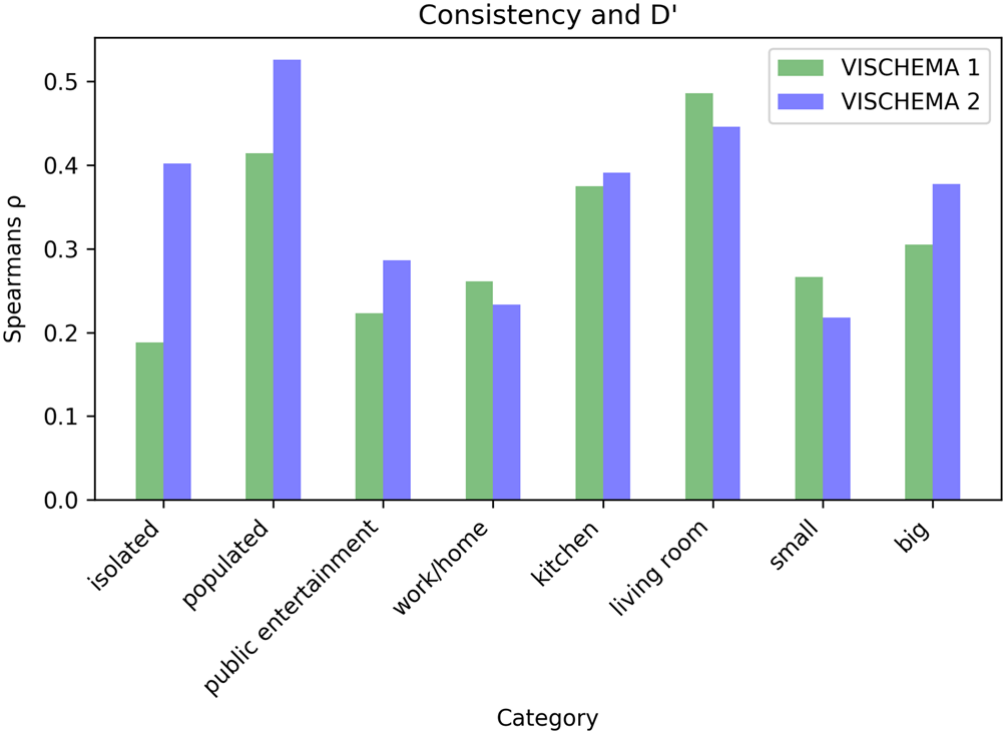
Histogram showing the correlation between per image category consistency for Vischema 1 and 2 datasets and human observers’ memory. Similar pattern of correlations between datasets indicates the reliability of using Visual Memory Schemas.

In order to test that visual memory schemas can capture image memorability we calculate the signal strength of the observers’ memory for the given images by using the sensitivity index, also known as the $D'$ (D-Prime) measure. The sensitivity index, $D'$ is a measure from signal detection theory that represents the strength of a given signal, in our case characterising the human observers ability to remember the given image. The results for the $D'$ scores are provided in Table 2 and similar to the consistency of memorable regions show that not all image categories are equally memorable. Strong overall positive correlation between image memorability measured with $D'$ and per category consistency of VMS maps for both VISCHEMA 1 ($\rho = 0.83$, ${\text {p}}<~0.05$), and VISCHEMA 2 ($\rho = 0.76$, ${\text {p}}<~0.05$) suggest a robust relationship between the two measures. When comparing this correlation for each image in each category, we also see a positive correlation, shown in Fig. 1. The overall high VMS consistency and positive correlation with the image memorability signal (measured by $D'$) indicates that VMS maps are a good descriptor of image memorability. In the following we refer to the combined VISCHEMA 1 and 2 datasets as the VISCHEMA PLUS dataset.

**Table 2:** D-Prime analysis of human memory for each category in the Vischema 1 and Vischema 2 datasets. High values clearly indicate that the memory signal for the given image category is strong and thus image memorability for human observers is high. Certain categories have stronger signals than others, possibly due to easier or more available encoding schemas for that category among the human participants.

Category	D Prime	
	VISCHEMA 1	VISCHEMA 2
Isolated	1.008	0.692
Populated	1.47	1.197
Public Ent.	2.037	1.813
Work/home	1.896	1.38
Kitchen	1.602	1.257
Living room	1.725	1.252
Small	1.52	1.4
Big	1.741	1.7

### Generating memorable images based on VMS maps

In our study we start by developing two different deep learning network architectures for generating memorable complex scenes, one based upon the Wasserstein GAN^19^ capable of producing images up to $128 \times 128$ pixels and the ProGAN architecture^20^ architecture capable of producing images of up to $256 \times 256$ pixels resolution. In order to generate images of varying level of memorability we explore incorporating the data driven VMS maps into the deep learning training and generation algorithm in two different ways. The first is by considering it as a single score while the second is as a spatial map constraint in the loss function used for training the deep learning models. We evaluated these two different constraints in the Wasserstein GAN architecture by assessing whether the newly generated images can produce a differential score when applying a computational single-score artificial memorability predictor. Our ProGAN-derived architecture is capable of generating images of a sufficient quality and resolution for human observer experiments, with which we validate our approach.

#### Single-score constraint

The first implementation of VMS maps in a Wasserstein GAN architecture is in a form of a single score that is based on the average intensity of the VMS map (i.e. observer consistency) and is used to modify the memorability of the generated image. We hence refer to our Wasserstein-based memorability generation network as W-MEMGAN. We generate a range of images characterised by various levels of memorability, from low to high, by fixing the generators latent code $\mathbf {Z}$, which controls the semantic content of the generated images, and varying the memorability input $\mathbf {M}$ to control the memorability of the generated images.

**Figure 2:**
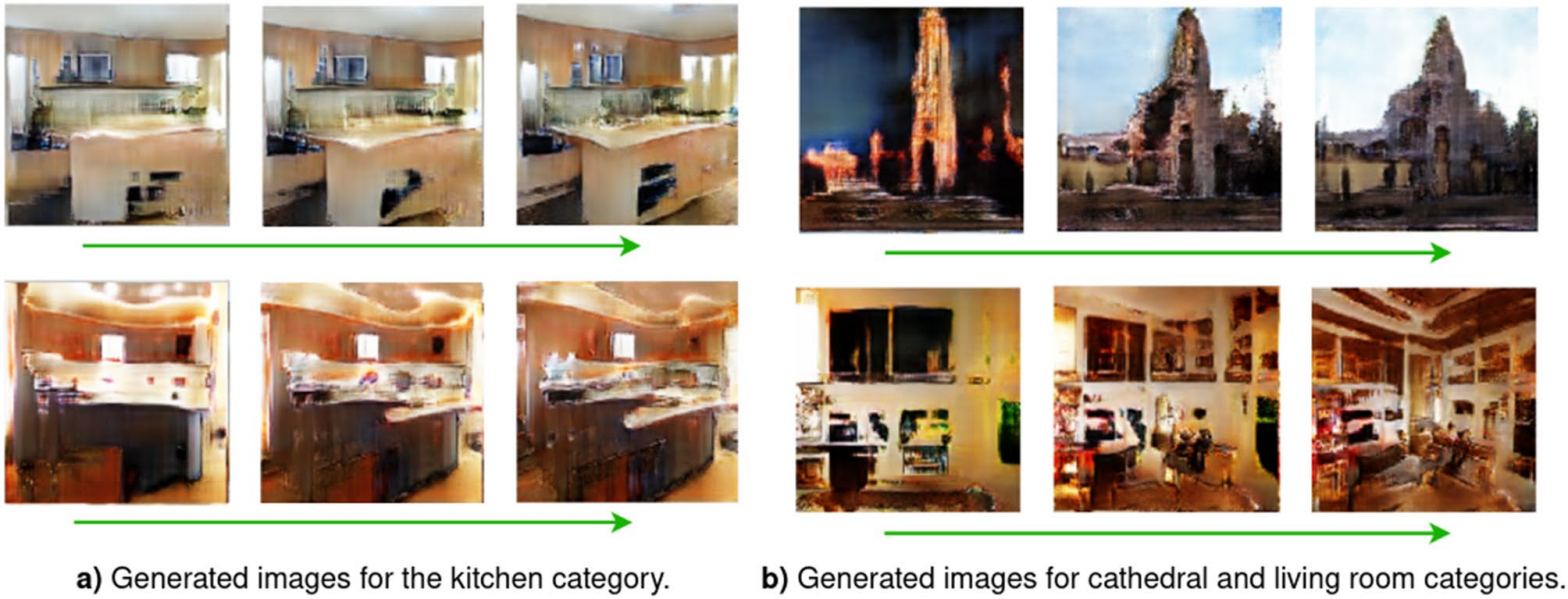
Generated images when fixing $\mathbf {Z}$, where the sequence of generated images is displayed from left to right, while the memorability $\mathbf {M}$ is varied from low to high. Shown categories include kitchens, cathedrals, and living rooms.

**Figure 3:**
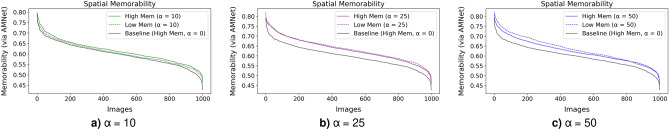
Predicted low and high memorability for different memorability weighting factors for $\alpha$, considering $\alpha = 0$ (in Eq. 3) as baseline. When increasing $\alpha$, all generated images have a higher memorability than the baseline. The most memorable images overall are obtained with $\alpha = 25$, but the best pairwise effect is achieved with $\alpha = 10$.

**Figure 4:**
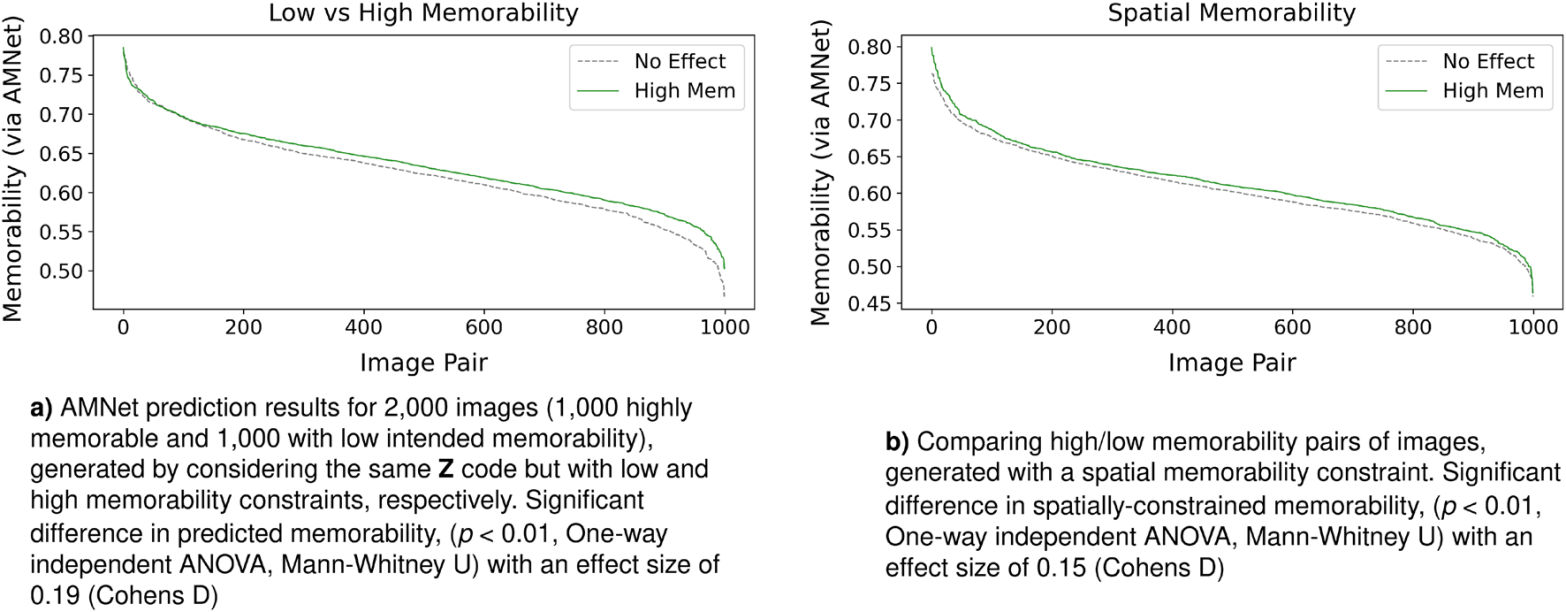
Differences in predicted memorability for low and highly memorable images generated with W-MEMGAN.

The newly generated images are created in ascending memorability in order to examine the variation space between exemplars of non-memorable and memorable images of a given image. Figure 2a,b show the generated images for different examples of scene from different scene categories, obtained by fixing $\mathbf {Z}$ while varying $\mathbf {M}$ from low memorability to high memorability. Just from visual evaluation of the images it is evident that clear differences emerge between images when increasing the memorability constraint. We can observe in all the scenes from Fig. 2a, that as memorability increases, semantic details and a more realistic ‘kitchen-like’ appearance emerges. The low memorability cases appear to display semantic ‘noise’ representing a collection of mismatched features with loose spatial relations. The less memorable images may display the typical elements of a kitchen, but lack structure, or rather the correct spatial relationship between the elements. It appears that by defining visual memory schemas as constraints of memorability results not only in the appearance of memorable semantic details, but also enforces spatial relationships between these details. This lends evidence that VMS maps capture semantic details and structures which match learned schemas held in human cognition. From Fig. 2b we can observe that when increasing the memorability, this results in a better image structure, clarity, and detail, resulting in images that better match human cognitive schemas (Figs. 3, 4).

In order to evaluate the newly generated images in a more quantitative fashion we generate 2000 images by setting the memorability constraint $\mathbf {M}$ either to very low or to very high. This results in the generation of pairs of images where only the memorability information varies between the two generated images while having the same random seed $\mathbf {Z}$. These images are then evaluated using AMNet^21^, an independent state-of-the-art memorability prediction network. AMNet predicts the memorability of images on a scale between 0 and 1.0, allowing us to calculate the difference between our population of intended memorable and non-memorable generated images, while also allowing us to inspect the difference between the newly generated paired images. The results in Fig. 4a show a statistically significant difference in memorability ($p < 0.01$) between the two populations. Images generated to be memorable clearly show a trend to be predicted as more memorable compared to the baseline population of low-memorability images. To note is that not all the highly memorable generated images are themselves equally memorable independent from the memorability modulation as we have seen when looking at memorability across different scene categories. Thus, when examining the pairs of our generated images, we find that as overall image memorability decreases, it becomes more difficult to influence the memorability of certain scene image categories which are already not particularly memorable. When the image generated to be memorable has a predicted memorability above 0.65, then 79.5% of the pairs of memorable and non-memorable images have a positive difference in memorability. When memorability falls below 0.65, only 40.7% of the pairs have a positive difference in memorability, where a ‘positive difference in memorability’ indicates that the image generated to be memorable is predicted as more memorable than the image generated to be non-memorable.

#### Spatial map constraint

A single score for an entire image does not capture spatial information about the memorability in the scene. As VMS maps reveal, not all regions of the image are equally memorable and in many cases memorability is concentrated on certain structures inside the image. We hypothesise that these carry semantic information that matches corresponding cognitive structures (schemas) used by the observers to encode and then retrieve information from long-term memory. It is highly unlikely that human cognition itself uses a single score to represent how memorable an image is, and whether it should be encoded. There are most likely multiple characteristics within an image associated and encoded with an episode of encountering that image. Instead, we hypothesised based on numerous findings from Cognitive Science that how closely a viewed scene corresponds to a cognitive schema plays a role in image memorability or rather how much is an image memorable to a human observer. While single score methods might be able to predict image memorability, they do not reveal anything about why the image is memorable for a human observer, or which elements in it cause that image to be remembered. We instead base this constraint on the concept of a visual memory schema represented in two dimensions; an organisational map of semantic elements shared amongst human observers that enable the encoding and recognition of scenes.

This method naturally lends itself to a two-dimensional representation of image memorability; the regions captured inside a visual memory schema map are thought to directly represent the semantic elements that lead to that image’s encoding and recognition. These elements correspond with schemas held in the brain; cognitive structures that represent the typical elements (and arrangement of elements) of a scene. A human, through life long experience and acquired knowledge, may construct a schema of a kitchen, learning that a kitchen may contain countertops, an oven, and kitchen appliances (this is an example; real schemas are likely more complex and flexible). Scene images that better match this mental schema in both arrangement and semantic presence have an encoding advantage against kitchen scenes that lack these elements or arrangements. Computational measures that employ visual memory schemas can be thought of as learning a method to replicate human scene memory that more closely mirrors the method the human brain uses to encode scene images.

Hence, to take advantage of the 2D characteristics of VMS maps, we modify W-MEMGAN to take as input a $10 \times 10$ pixel map describing the intended spatial memorability of the generated image. The provided input are artificial VMS maps created using a deep learning method trained on VISCHEMA 1 and 2 (VISCHEMA PLUS), similar to those obtained from human observers.As with single-score VMS constrained memorability, employing artificial 2D VMS maps to alter the memorability of generated images also results in a statistically significant difference between populations of 1,000 generated memorable and 1000 generated non-memorable images, shown in Fig.  4b. These findings indicate that both single-score and spatial constraints extracted from the VMS maps incorporated into our W-MEMGAN architecture are capable of modulating the memorability of newly generated images evaluated by an artificial memorability predictor such as AMNet. The non-spatial single-score implementation of the VMS results in a greater effect size of the difference in memorability (0.19 vs 0.15, Cohens D) compared to the spatial method. We postulate that this could be the result of additional difficulty of integrating a spatial constraint compared to calculating a single score constraint for the entire image.

To examine the effect of the feedback strength of the memorability feedback mechanism we tested several different values for $\alpha$, the hyper-parameter which controls the ‘strength’ of the mechanism and defines how strongly we intend memorability to affect our generated images. The effect of four different values of $\alpha$: 0, 10, 25, and 50 on the prediction of low and high memorability in generated images is shown in Fig. 3). For $\alpha =0$, the memorability predictor provides no feedback to the network, disabling the influence of VMS maps and hence is used as a baseline. Best results are achieved for $\alpha =10$, resulting in the clearest difference between high and low-memorability images and noticeably above those of the baseline, Fig. 3a. Using an $\alpha = 25$ resulted in generation of images with high memorability scores but with reduced ability to discriminate between the low and high memorable exemplars. Higher values for $\alpha$ prevent the W-MEMGAN from distinguishing between high and low memorability images, instead just raising the memorability of every image generated by the network, as shown by the results from Fig. 3b,c.

### Human memory performance for generated images

**Figure 5:**
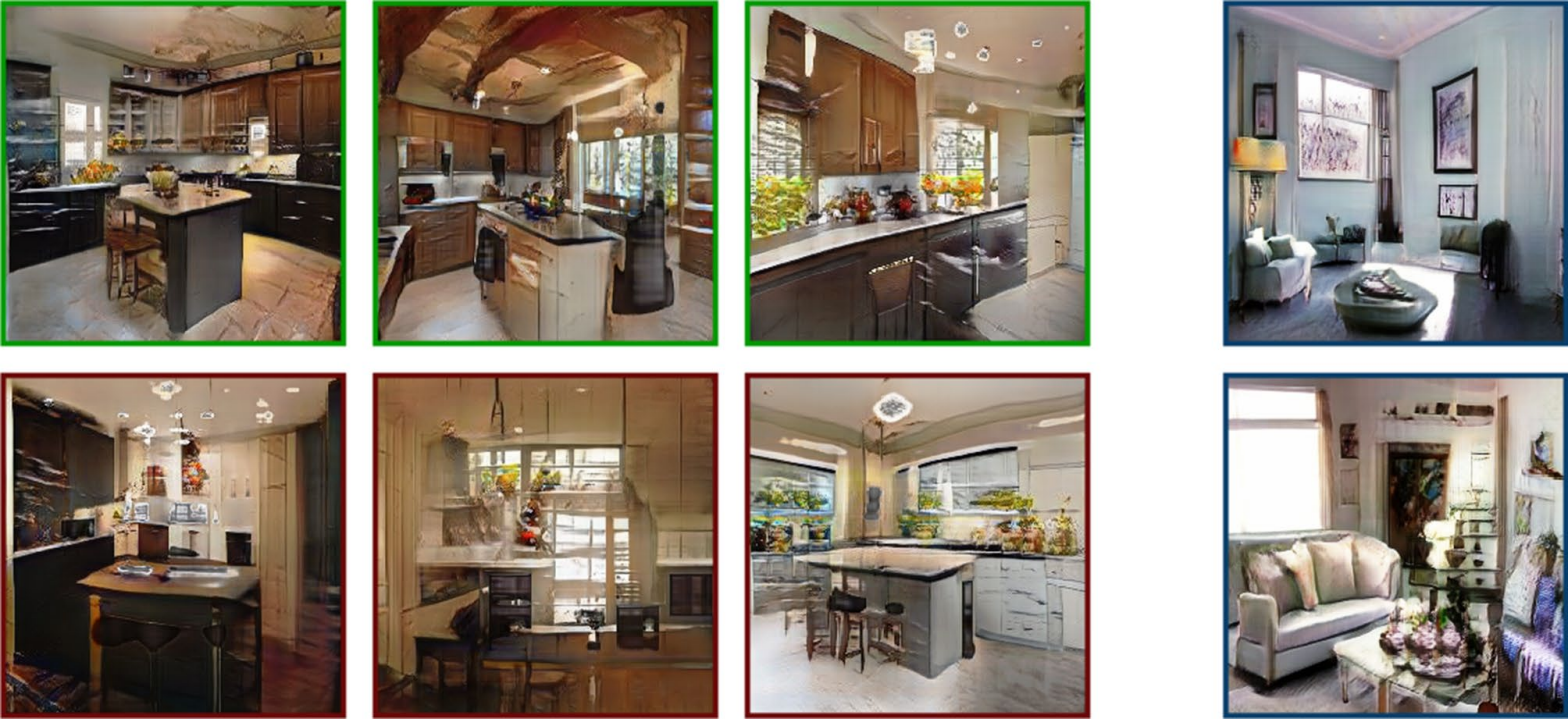
Memorable, shown within green boundaries and non-memorable, shown within red boundaries generated image pairs. Foils are shown within blue boundaries.

**Figure 6:**
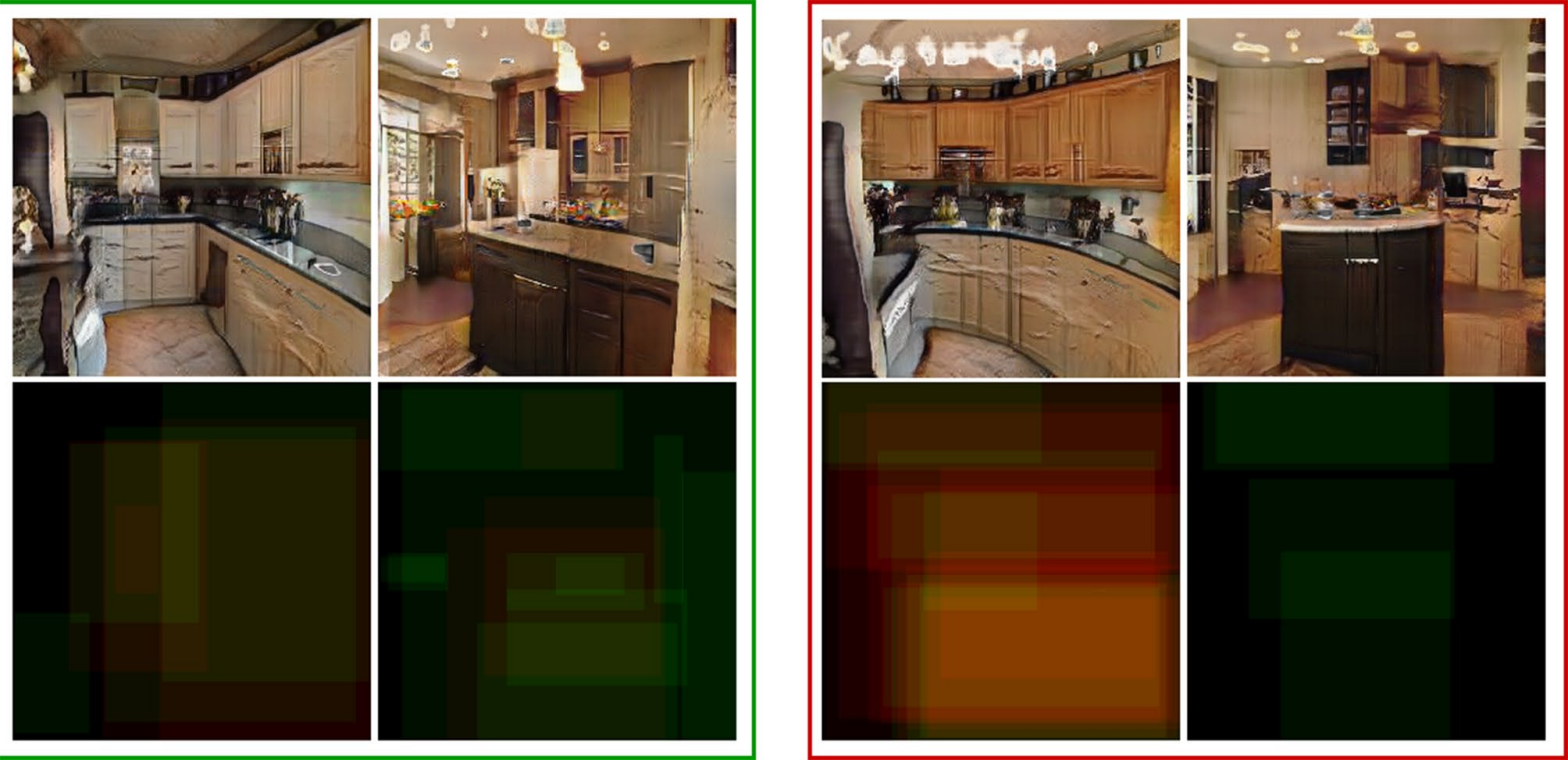
Generated high-memorability images (left) and their low-memorability pairs (right). VMS maps for each image are shown on the bottom row.

We test the feasibility of directly modulating image memorability using VMS maps by conducting a visual memory experiment with human observers. The images used in the experiment were generated by our second architecture; based upon ProGAN^20^ combined with memorability feedback, which we term ‘MEMGAN’. MEMGAN enables the creation of higher resolution images at a much higher quality than the W-MEMGAN architecture. Based on our previous results, we weight the memorability constraint to a value of $\alpha =10$, which gives the best partition between memorable and non-memorable generated images. Examples of images generated are shown in Fig. 5. In the experiment, human observers were asked to view a stream of generated images presented for 3 s each one at a time. Participants were asked to recognize images they recognized as repeats and indicate upon identifying a repeat the areas in the image that made them remember the image. This allowed us to evaluate the memorability of generated scenes through the hit rate of the images (how often an image was successfully recognized as a repeat) and the consistency of the VMS maps across observers (regions in the image indicated as memorable areas, see the examples from Fig. 6).

To determine how consistent our participants were with each other, we take 25 equal splits of our visual memory schema map and hit data for each split, and then compare them against one another. We find a hit-rate consistency of 0.3 (Spearmans $\rho$, $p<0.0001$ for all splits) and an overall VMS map consistency of 0.38 (Pearson Linear Correlation Coefficient). The VMS map consistency is lower than the 0.67 presented in^12^, but this is expected given that our task contains only two different categories of images, and is thus a very homogeneous stimulus set. Nonetheless, there exists a clear consistency between participants.

#### Differences in observed memorability for generated images

**Figure 7:**
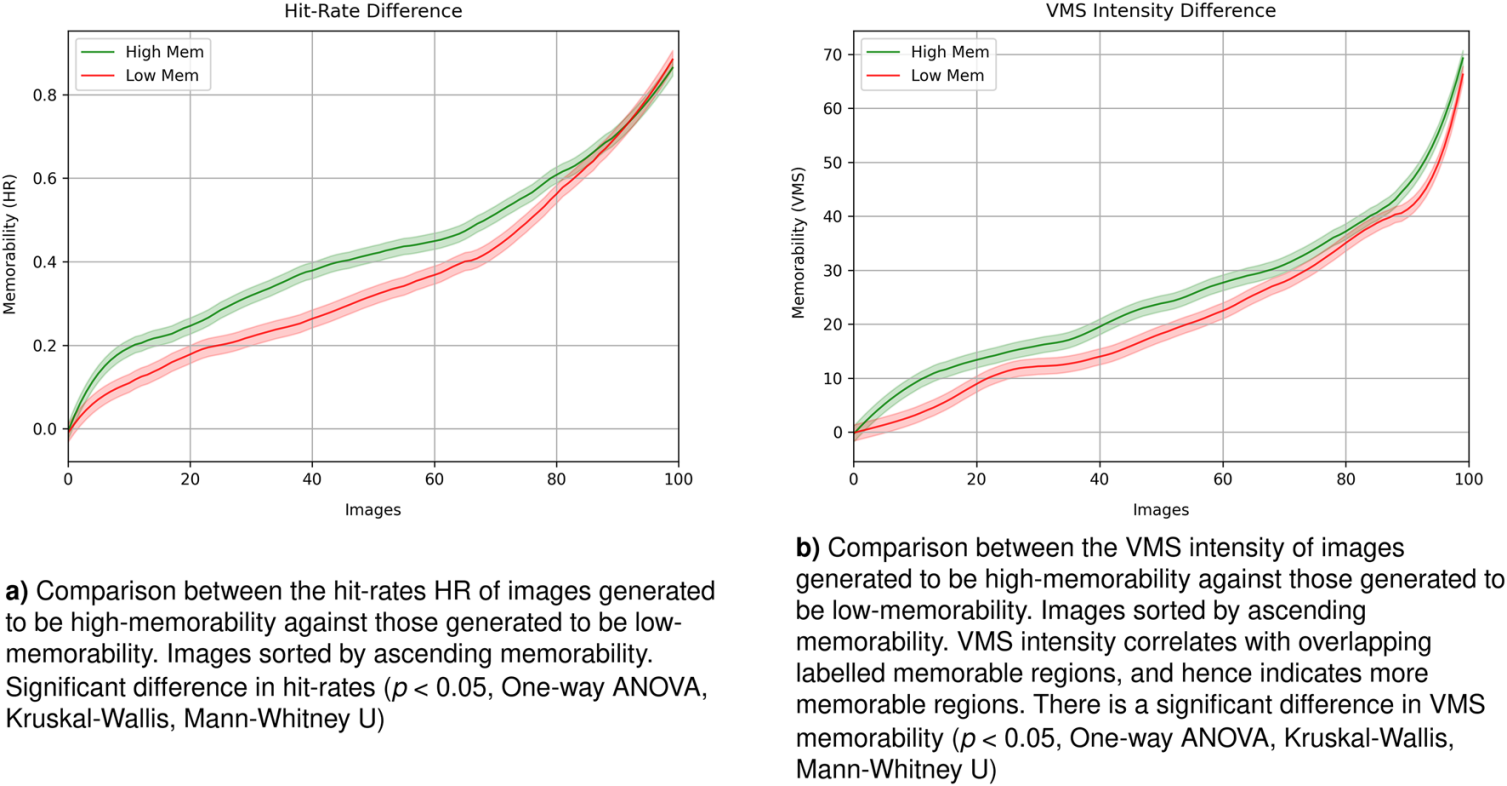
Difference in memorability (HR and VMS Intensity) for generated image populations. Degree 3 polynomial fitted for visualisation.

The evaluation of the hit rates for the generated high and low memorable images (Fig. 7a, shows that high memorable images result in both higher hit rates (0.45 for high and 0.39 for low memorable) and higher false alarms rate (0.20 for high and 0.16 for low memorable). However there is a statistically significant difference ($p < 0.05$) for the hit rates but not for the false alarms rates between generated images as highly memorable and those with low memorability. This pattern of results, of a robust difference in hit rates and a lower difference in false alarms, is expected^13,22^ given that the same structures that enable easier encoding of a scene, also make it more likely for a human to believe they have seen that scene. Indeed, within the VISCHEMA image set with which the memorability evaluator was trained, a rise in memorability corresponds with a rise in false memorability ($\rho = 0.19$, $p < 0.001$).

#### Differences in observed visual memory schemas for generated images

Comparison of differences in Visual Memory Schemas between high and low memorability images required that we first condense each VMS map down to an average intensity. Outliers beyond two standard deviations of the mean were excluded. This gives us separate values for both images that were correctly recognised as seen before (true memorability) and those misrecognised (false memorability). There is a statistically significant difference ($p < 0.05$, effect size 0.36 Cohens D) in the VMS memorability channel for highly memorable images vs low memorable images, with a robust Bayes factor $ln(BF) = 1.117$ indicating substantial evidence for the effect of modulating memorability (see Fig. 7b. As before, there is no statistical difference for false memorability between image catgeories. We also compared predicted VMS maps from the generator network to those outlined by the human observers and found a Pearsons Correlation of 0.49 ($p<0.05$), a Spearman rank correlation of 0.5 ($p<0.05$), with a population average mean-squared error of 58 between predicted and human-gathered VMS maps. These results indicate that the relational memorability patterns used to generate the images are effective in defining visual memory schemas in the same images, which correlates positively with those indicated by human observers.

#### Image pair analysis

The results for both the hit rate and observed VMS’s are encouraging, and clearly show a statistical difference between the overall populations of highly memorable and low memorability generated images. However, the images were generated in pairs, with high and low memorable versions of the same scene image, as defined by a fixed latent code, and by modulated memorability, and thus can be compared as such. This requires the evaluation of the pair-wise difference, between an image with the same latent code but modulated memorability. Results indicate a statistically significant difference ($p<0.02$, paired T-test, Wilcoxon signed-rank test) for both hit rates and VMS memorability.

#### Comparing our results with an independent computational predictor of memorability

We also compare the results obtained on our human observer study with those of a recent state-of-the-art memorability predictor^21^, trained on the same dataset (LSUN^23^) from which we drew the training data for our MEMGAN models. We find no significant correlation between the memorability scores calculated by the independent memorability predictor for our images with the experimentally obtained hit rates or VMS map intensity of our images. This finding suggests that memorability predictors based only on single-score models of memorability are missing important characteristics of human visual memory, and that state-of-the-art predictors fail to predict human memory performance for generated images. However, we do see a significant effect ($p<0.05$, Paired T-test, Wilcoxon signed-rank, Mann–Whitney U), when comparing paired predicted scores for our generated high and low memorability images, which suggests computational predictors can differentiate between populations of generated images.

## Discussion

In this research study we present and evaluate a method of generating scene images constrained by a construct from cognitive science: visual memory schemas, and test its validity to modulate human episodic memory of images. The modelling of the VMSs is data driven and based on human memory study. We directly manipulate the visual schemas of images in a generative deep learning model (MEMGAN) and hence influence the final memorability of generated images. To our knowledge this is the first example of a generative model specifically trained from scratch to generate memorable scene images employing two-dimensional memorability data gathered from human observer experiments. Moreover, we double the size of an existing two-dimensional memorability dataset, and for the first time investigate the relationship between VMS map consistency and image memorability, along with presenting per-category consistency data for VISCHEMA categories. Encouragingly, consistency values remain high for both the original VISCHEMA dataset and our second replicated experiment, confirming the validity of this approach of gathering two-dimensional memorability maps.

There is currently a limited number of existing approaches to the problem of modification of image memorability. Sidorov et al.^16^ examine various methods for altering the memorability of images, from basic-photo editing techniques such as adjusting the saturation of the image, to the employment of an attention-based Generative Adversarial Network (GAN) for generating memorable face photographs. The memorability data used as input for training the GAN was drawn from artificial memorability predictors. They find that both their altered and generated images produce changes to the artificially predicted memorability score of images but do not have any data on human observers. This approach is similar to that from^17^, in which a deep style-transfer model was trained to automatically apply ‘filters’ such as sepia tones or saturation boosters to images in order to boost said images memorability. In^10^, Lore et al. develop a transformer network that can be attached to an existing generator in order to adjust the memorability of generated images. While this approach does leverage existing trained networks to generate photorealistic images, this is dependent upon the chosen generator, and additionally requires a generated ‘seed’ image for the network to adjust. As a feedback mechanism they employ single-score memorability predictors. They show through human recognition trials that the images adjusted to be more memorable tend to be empirically more memorable.

Prior approaches to memorability modification require a starting image (real or generated), and it is the memorability of this image that is then modified. We instead desire to create an approach that can generate images without requiring this initial seed image, and can instead synthesise recognisable scene images given only a latent code and a desired memorability. As proof of concept that cognitive relational patterns can serve as the basis for a generative network for memorable scene images we develop and train an architecture that can synthesise low-resolution memorability-constrained images. We evaluated the potential of both single-score VMS based memorability and spatial memorability as a driving mechanism for scene image generation. The generated images, despite low resolution and relatively poor quality, are capable of causing a significant effect in a state-of-the-art third-party memorability prediction network that had never previously seen the generated images. Further, by modifying the strength of the memorability feedback mechanism our memorability constrained images can be made to display both higher and lower image memorability compared to a baseline of generated images where the memorability feedback network is disabled. Interestingly, placing too much emphasis on the feedback network causes the network to lose discriminative power, becoming unable to correctly generate images with high vs low memorability, yet generating images that were predicted to be much more memorable overall. This provides evidence that we can manipulate the memorability of generated images in a meaningful way. Image memorability based on VMS maps appears to control both the emergence of semantic details as well as the spatial relationships created between these details.

The final test of visual memory schemas as viable mechanisms for modulating image memorability is whether our approach could functionally work with actual human observers and not only with computational memorability predictors. By integrating our memorability evaluator and loss component with a more advanced generator allowed us to influence the memorability of relatively high resolution, high-detail images. While we lack the resources to generate high resolution photo-realistic images, the images we do generate show clear structure, detail, and are certainly recognisable as belonging to their intended category. From our results (and given that we based our model constraint on visual memory schemas), we hypothesise that more memorable scenes better match the cognitive schema of that scene contained within the human mind. In this work, we observe that making a scene more memorable results in changes to the structure and content of the generated scene compared to the same scene generated to be of a lower memorability. We hypothesise these differences cause the image to become closer or further from the mental representation (i.e, the schema) of that scene which is stored within the human brain. However, it is unclear whether the differences in schema between high and low memorability images also result in greater difficulties visually recognising the image as an exemplar of its class. To test this, we employed a scene recognition deep neural network (ResNet152^24^) trained on the Places365 dataset^25^ to categorize every synthesised image. We find that in general the majority of images are classified as their class (or a highly similar, related class, e.g., galley vs kitchen). For highly memorable generated images, 96% of images are correctly classified and for low memorability generated images, 95% of them are correctly classified as kitchens by the scene recognition network. There appears to be little difference in how visually recognisable the generated images are as members of their class; and the demonstrated memorability effect appears independent of visual recognizability. Given that human ability to categorize scene images generally exceeds that of neural networks; the recognizability scores shown are best viewed as a lower bound. Additionally, as human memory is not contingent upon resolution^26–28^ and perfect photo-realism, the images we generate serve well for their intended purpose. We show through human observer memory experiment that the images we generate to be more memorable are more likely to be detected correctly as repeated images by humans. Additionally, we find that the false-alarm rate of said images also increases, an encouraging sign that our images are truly modulated by visual memory schemas, as the exact same effect appears in the VISCHEMA dataset of real images. The cognitive schemas that aid the remembering of scenes also lead to false remembering when presented with a memorable image modelled on the schema, even if that image has never been seen before. Critically, we are able to generate memorable images without requiring a seed image, such as the approach employed in^10^, and verify that two-dimensional maps of memorability can be employed to modulate memorability, rather than relying on single-score approaches.

In summary, our results indicate we were able to both fool computational memorability predictors, and manipulate human visual long-term memory via artificially generated images, constrained with a two-dimensional visual memorability schema concept borrowed from cognitive psychology, for which there are neural correlates^29^. It may appear circular that we have constrained a model with visual memory schemas, (which indicate memorable regions) and find that our generated images are indeed memorable. However, this only appears this way because the data shows an effect on human memory; there was no guarantee that this was possible to accomplish. There is additionally no guarantee that the generative model would be able to be constrained by the visual memory schemas. There is little work in this area (and none that examines the visual schemas of generated images); and in essence the model is the test—investigating whether it is, or it is not possible to use visual memory schemas to synthesise scenes that can modulate human memory. We find that by employing VMS maps we are able to generate completely new artificial scenes that cause a desired modulation of human memory as tested by a human observer memory experiment. This has interesting implications for the future study of image memorability, as well as real-world applications for memorability research.

We have designed a neural network that appears to understand visual memory schemas to a sufficient enough degree to use them to visibly change the output of a generated scene, based upon a brand new, extrapolated or invented schema (of controllable memorability), that we want the scene to match. The generative network is constrained by an artificial VMS map predictor that can produce two-dimensional memorability maps for arbitrary scene images; the greater the difference between the predicted VMS map and the target VMS map for a synthesised scene, the more the network is penalised. As we have shown, when we constrain a synthesis network with a VMS predictor, we find that we are able to generate scenes that affect human memory for those scenes, or rather, affect their the performance on a memory test. We learn from this that visual memory schemas appear a strong enough descriptor of what information humans encode into memory to enact visible changes on the synthesised images based upon the input schema.

## Methods

### Obtaining visual memory schemas maps for 2D memorability prediction

Recent work employs human observer experiments to capture the regions in the image that cause said image to be remembered or falsely remembered^12^. This dataset is known as VISCHEMA, and contains 800 images and 800 visual memory schema maps. These visual memory schema maps correspond to mental knowledge frameworks employed by humans to encode the said image into memory, and enable retrieval later. Visual memory schemas (VMS) are hypothesised to relate high level image structures to the concept of memorability. These VMS maps were gathered via a behavioural experiment with human observers on a dataset of 800 scene images from eight different categories. Participants (n = 90) were asked to remember 400 images during a study phase. Then, during a test phase, they are shown 200 repeats and 200 non-repeated images, and asked to indicate whether they had seen an image before. The participants were also asked to draw boxes around the regions of the image that caused them to remember said image. When the image was correctly remembered, this produced ‘true memory schemas’ and ‘false memory schemas’ when incorrectly remembered. These VMS maps were shown to be highly consistent (correlation histogram mean of $\rho = 0.7$). VMS internal consistency is higher than both VMS correlations with eye fixations ($\rho = 0.5$) or saliency ($\rho = 0.58$). VMS-based results hence outperform simple metrics for predicting image memorability for the dataset used in the experiment. Progress has been made towards reconstructing these VMS maps artificially, both with fully convolutional models and variational models^13^.

**Figure 8:**
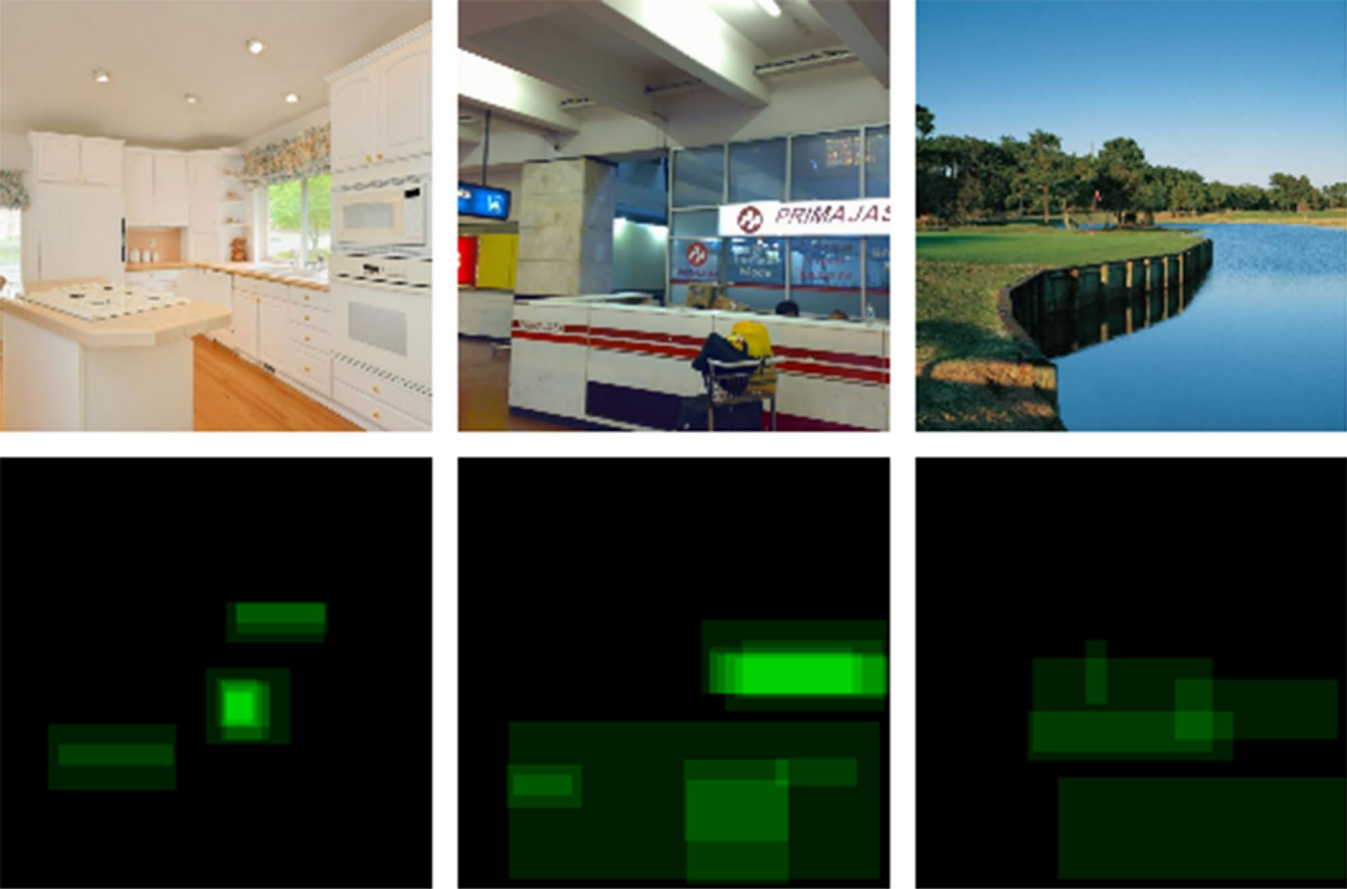
Images from VISCHEMA 2 dataset are shown in the top row, while in the bottom row are the image regions indicated by observers as causing them to remember the images from above. Source images are drawn from the public SUN dataset^30^.

For the purposes of this study we extend the original VISCHEMA stimulus set with additional data which we term VISCHEMA 2, examples shown in Fig. 8. The VISCHEMA 2 dataset contains an 800 new images and 800 VMS maps of the same categories as VISCHEMA 1^12^, gathered through human observer experiment, with 60 participants, thus creating together a 1600 image/VMS pair dataset referred to as VISCHEMA PLUS. This dataset is used to train the auxiliary loss function in our generative approach and used to produce the new memorable images. VISCHEMA data is available at^18^. The experiment to gather additional data was approved by Departmental Ethics Committee of the Dept. of Psychology, University of York, UK, and follows the relevant guidelines given by that committee. Informed consent was obtained from participants, and they were free to withdraw at any time.

### Memorability estimation feedback network

The assessment of image memorability is performed by employing a Visual Memory Schema prediction model developed in^13^, which is based on the Variational Autoencoder (VAE)^31^ learning model. A VAE is made up of two convolutional networks: the encoder aiming to extract a latent space representing the data, and the decoder which aims to reconstruct the given data. Following training, given an image, the VAE is used to predict its corresponding VMS map. We train this model on the VISCHEMA PLUS dataset containing 1600 image/VMS pairs. The output of this model is a two-dimensional VMS map. This predicted VMS map is based upon the latent space of the VAE, which corresponds to a learnt mapping of image features to memorability based upon multiple human observations for the input image. We only consider the ‘memorability’ channel of the VMS maps (true schemas), and do not make use of the ‘false memorability’ (false schemas) information. For the given VMS data $(\mathbf{x}$, the encoder of the VAE infers a latent space $\mathbf{z}$, by using the following loss function:

1 $$\begin{aligned} L(\theta ,\phi ) = -E_{\mathbf{z} \sim q_{\theta }(\mathbf{z}|\mathbf{x})}[\log p_{\phi }(\mathbf{x}|\mathbf{z})] + KL(q_{\theta }(\mathbf{z}|\mathbf{x})||p(\mathbf{z})), \end{aligned}$$

where the former term represents the log-likelihood of VMS reconstruction by using the decoder network and the latter represents the Kullback–Leibler (KL) divergence between the variational distribution $q_{\theta }(\mathbf{z}|\mathbf{x})$ and the prior $p(\mathbf{z})$ aiming to assess the image reconstruction ability of the network. $\theta$ and $\phi$ represent the parameters of the VAE’s encoder and decoder networks, respectively.

### W-MEMGAN architecture and training

**Figure 9:**
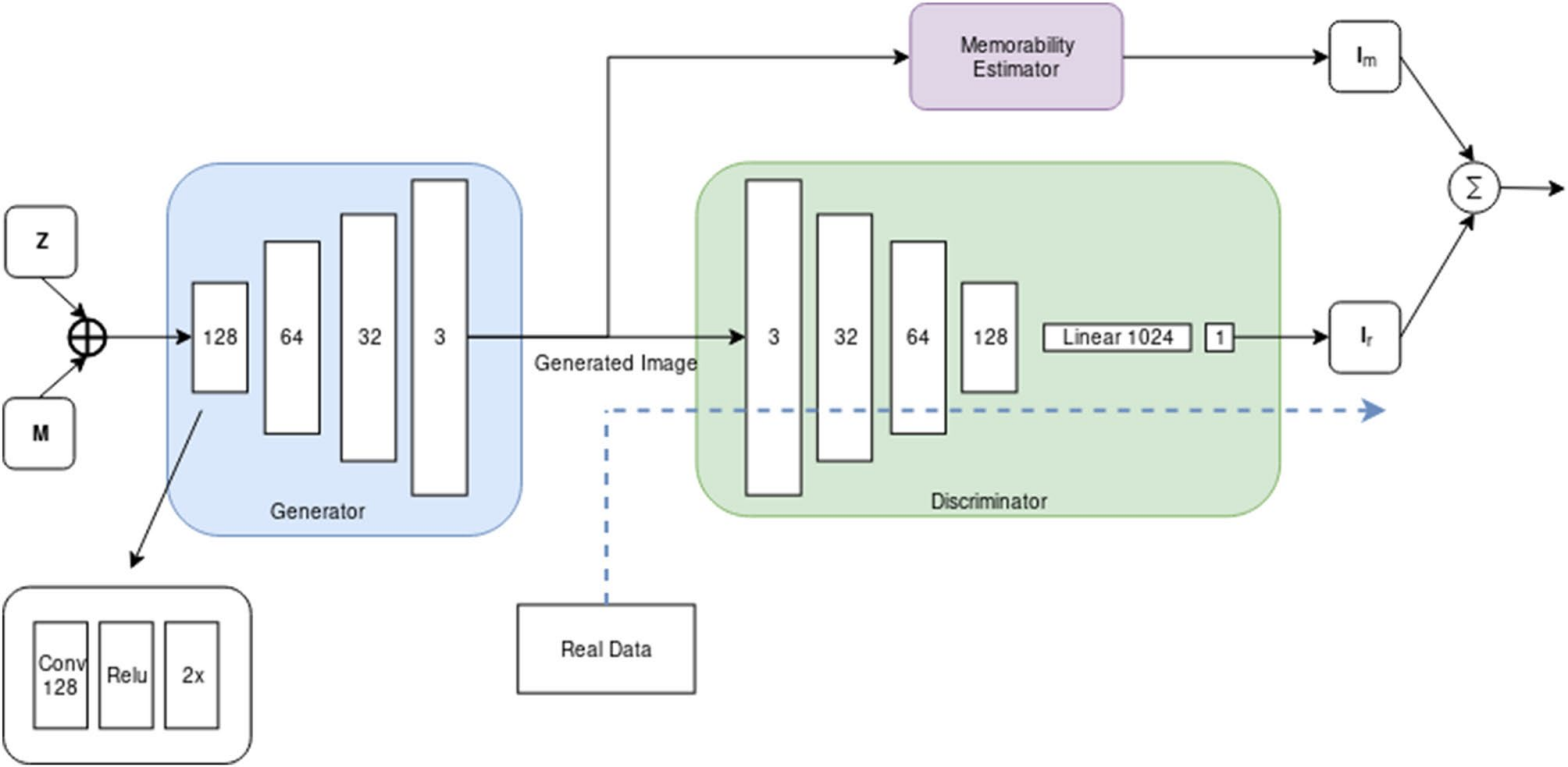
Memorability-constrained image generation model architecture. PixelNorm and Minibatch Standard Deviation layers omitted for clarity.

The diagram of the deep learning architecture used for generating memorable images is shown in Fig. 9. It consists of a Generator *G*, a Discriminator *D*, and the memorability feedback network $\mathcal{M}$. While the generator creates memorable images, the discriminator evaluates the ‘realness’ of the generated images, and the auxiliary memorability network evaluates whether the memorability of the generated image matches the memorability defined by a memorability constraint $\mathbf {M}$. $\mathbf {M}$ in this case may either be a two-dimensional target VMS map, or a single target memorability score. The image generation network *G*, corresponding to the generator from WGAN, aims to synthesise an image $\hat{\mathbf {I}}$ using random variables $\mathbf {Z}$ as inputs, which defines the latent space of the MEMGAN, while $\mathbf {M}$ acts as the memorability constraint:

2 $$\begin{aligned} \hat{\mathbf {I}} = G(\mathbf {Z}, \mathbf {M}). \end{aligned}$$

The output of the generator is a generated image $\hat{\mathbf {I}}$, whose memorability score is as close to $\mathbf {M}$ as possible. Both $\mathbf {Z}$ and $\mathbf {M}$ are drawn from Gaussian distributions. The generator is constrained by both the discriminator *D* and by the memorability feedback network $\mathcal{M}$, which estimates the memorability map $\hat{\mathbf {I}}_m = \mathcal{M}(\mathbf {I})$. The discriminator *D* is implemented as an improved Wasserstein GAN model^32^ which employs a penalty term on the discriminator loss yielding better performance and stability when compared to the classical GAN^33^.

During the training, $\mathbf {Z}$ is sampled randomly from a Gaussian distribution, and $\mathbf {M}$ is either sampled from the Gaussian distribution or $\mathbb {P}_{t}$, the distribution of possible target VMS maps, depending whether the network is being trained for spatial memorability or single-score memorability. When training the discriminator *D*, $\mathbf {M}$ is discarded, as it is only necessary for training the generator, where it is used to calculate the memorability loss. This has the effect of penalising the generator if the generated images are not of a similar memorability to that defined by $\mathbf {M}$. For example, if the image was intended to be memorable while actually it is not memorable, the generator loss will increase. Each training epoch consisted of 60,000 kitchen images drawn from the LSUN database^23^, and the network was trained for 500 epochs, which took approximately 8 days on 4$\times$ Nvidia 1080 Ti GPUs.

### MEMGAN architecture and training

The Wasserstein GAN based network does not generate memorable images at a sufficiently high resolution and quality for human trials. Given the latent code $\mathbf {Z}$ and an artificially generated target 2D visual memory schema (VMS) map $\mathbf {V}$, the goal was to generate a sufficiently realistic $256 \times 256$ pixel image from $\mathbf {Z}$, whose VMS is close to that of $\mathbf {V}$. We hence combine the memorability feedback network with a more suitable generator architecture, that of the progressive GAN^20^. We draw $\mathbf {V}$ from $\mathbb {P}_{t}$, the possible target VMS maps.

**Figure 10:**
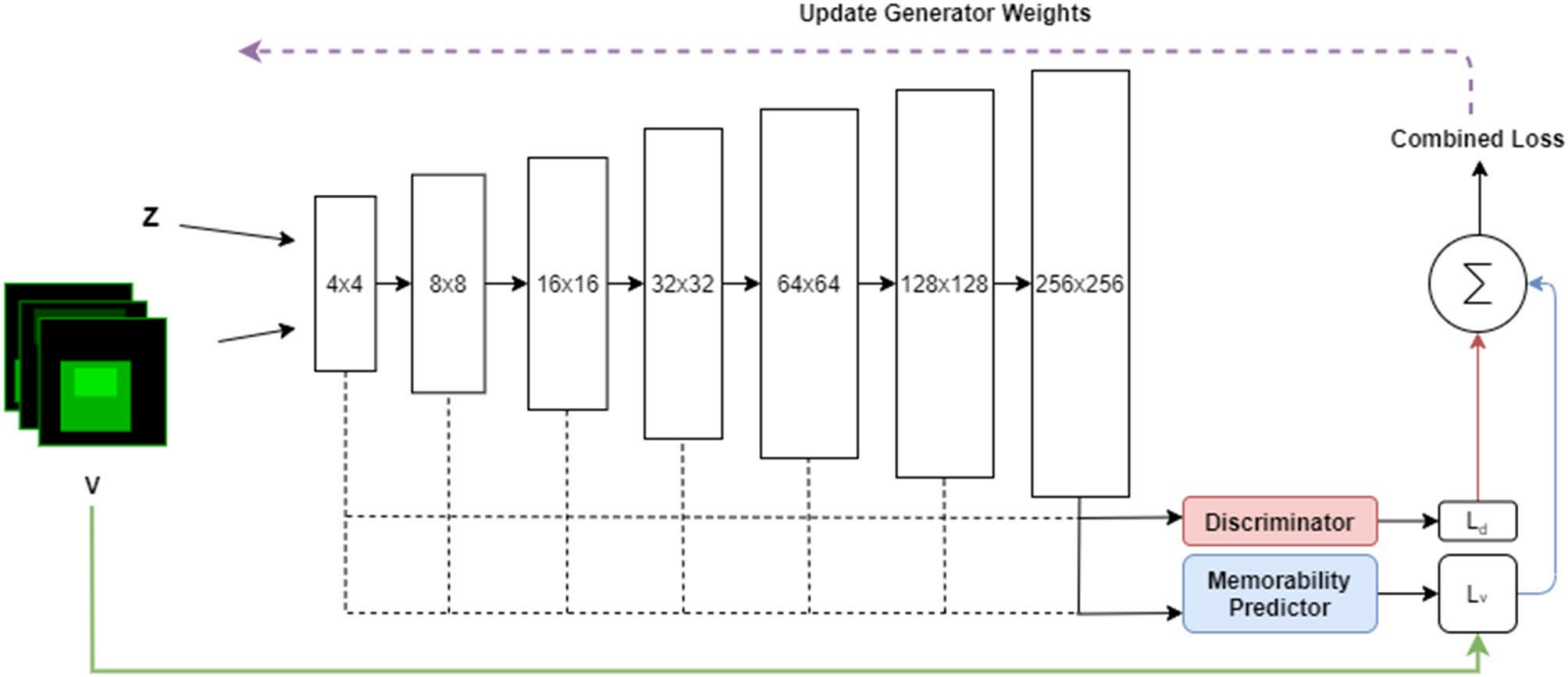
Progressive generator with per-resolution memorability estimation.

While aiming to obtain photo-realism is preferable, it is not a strong requirement for our architecture and subsequent human observer experiment. As long as the image generated is recognisably as a member of its target category, a human observer will employ the correct visual schema when encoding the image into memory. This allows us to reduce the capacity of the network compared to the original progressive GAN^20^, which results in an accelerated training time on the available hardware. A simplified (for visualisation purposes) architecture is shown in Fig. 10. The MEMGAN architecture we develop bears superficial similarities to both ACGAN^34^ and InfoGAN^35^, though rather than predicting discrete class labels or extracting interpretable dimensions in an unsupervised fashion, it generates memorable images, without a prerequisite seed image (such as those used in^10^), while being supervised by human observer-based cognitive structures. The generative network architecture has specific processing blocks for each image resolution, as can be observed in Fig. 10. The output image of each resolution block is passed through the memorability predictor as the network generates more accurate images of increasing resolution. As each resolution block takes over the information produced by the previous layer of processing blocks, the connection of those blocks to the memorability predictor is dropped. This allows the memorability signal to affect all resolutions of the generator during training. We only generate up to a resolution of $256 \times 256$ to limit the computation time, which is ever increasing when attempting to generate images of higher resolutions. The training time is reduced at the cost of losing some detail by reducing the capacity of the $256 \times 256$ and $128 \times 128$ resolution blocks by half. Finally we add a $tanh$ activation function at the output, before merging different resolution blocks, which aids stability.

We trained two deep generative networks in order to generate images for our human memory experiment, one with a memorability constraint (MEMGAN) and another without, whose purpose was to generate foil images. Both networks were trained for 200 epochs. Each resolution block was slowly introduced to the network over a duration of ten epochs, and then trained for an additional ten epochs before the next resolution block was introduced. Each of the first five resolution blocks of $128 \times 128$ pixels from Fig. 10, was shown a total of 4,800,000 images. The final block of $128 \times 128$ was shown 2,400,000 images. These images were drawn from a dataset of 240,000 kitchen scene images, and the same number of living room scene images, both drawn from the LSUN database^23^. This allowed a suitable balance between resolution, quality, and required total training time. We follow the example set in^20^ and use an Adam optimiser^36^ with the following parameters: $Lr = 0.0015$, $\beta _{1} = 0$, $\beta _{2} = 0.99$, $e = 1\times 10^{-8}$. Each of the two networks was trained for 14 days on 4$\times$ Nvidia 1080 Ti GPUs.

### Loss functions

Both our memorable image generators are designed to use the same loss function, the Wasserstein metric combined with a component which calculates the difference between the desired and generated memorability for a given image. This training mechanism works for both single-score and VMS map memorability training examples.

3 $$\begin{aligned} L = \underset{\hat{\mathbf {z}} \sim \mathbb {P}_{z},\mathbf {v} \sim \mathbb {P}_{t}}{\mathbb {E}} [ D(G(\hat{\mathbf {z}, v})) ] - \underset{\mathbf {x} \sim \mathbb {P}_{r}}{\mathbb {E}} [ D(\mathbf {x})) ] + \lambda Loss_{gp} + \alpha \underset{\hat{\mathbf {x}} \sim \mathbb {P}_{g}, \mathbf {v} \sim \mathbb {P}_{t}}{\mathbb {E}}[(\mathcal{M} (\hat{\mathbf {x}}) - \mathbf {v})^2] \end{aligned}$$

The loss function is designed to embed a memorability predictor and contains the following components: a generator network $G$, a discriminator $D$ and memorability predictor network $\mathcal{M}$. Considering the latent code distribution $\mathbb {P}_{z}$, target VMS distribution $\mathbb {P}_{t}$, real image distribution $\mathbb {P}_{r}$, predicted VMS distribution $\mathbb {P}_{v}$, and generated image distribution $\mathbb {P}_{g}$ based upon the latent code $\hat{\mathbf {z}}$ and $\hat{\mathbf {v}}$ we define the loss function in Eq. (3). The latent code $\hat{\mathbf {z}}$ is drawn from a Gaussian distribution and $\mathbf {v}$ from a distribution of target VMS maps, where height, width, and intensity of VMS regions is drawn from a uniform distribution. $\lambda Loss_{gp}$ refers to the gradient penalty loss in^37^. $\alpha$ controls the strength of the memorability loss. $\mathbb {P}_g$ represents the probability of the generated data and $\mathbb {P}_r$ is the probability of the real data. The additional term controlled by the hyperparameter $\lambda$ prevents the gradients inside the discriminator from violating Lipschitz continuity, whereas the first two terms evaluate the Earth-Mover distance between the generated and real distributions. The additional memorability loss, combined with the Wasserstein loss, constrains the image generation by both ‘realness’ and memorability simultaneously.

### Generating images for human observer experiments

We generated low-memorability and high-memorability kitchen images with our MEMGAN. To avoid making the task too difficult, we also generate memorability-unconstrained images of another interior scene category to act as foils in the memory experiment, living rooms. Our target memorability-modulated images are generated in pairs, with a fixed latent code Z per pair, varying the desired target VMS map between low and high memorability (modulating memorability constraint **M**); for each highly-memorable image there is a non-memorable image generated defined by the same latent code. We generated several hundred pairs and additionally memorability-unconstrained images as foils. Foil images and target image pairs suffering from extreme distortion were excluded from this study to avoid differences in memorability being caused by drastic quality differences between categories. In order to avoid any bias in the selection of images, if one image of a target pair is of acceptable quality to be included in a human trial, then the other image of the pair is automatically included as well. What is more there is little chance of biasing the images for memorability one way or another, as it has been shown in prior studies that humans cannot intrinsically predict the memorability of any given image^5^.

We selected 100 pairs of high and low-memorability generated images, for 200 memorability-constrained images overall. We additionally selected 200 generated living room scene foils of suitable quality. The resulting 400 images were used as a stimulus set for the human observer memory experiment that tested the validity of our memory modulation. We quantified the image quality differences by employing the Fréschet Inception Distance (FID)^38^ and note minimal differences between categories. The high-memorability images have a FID of 108 and the low-memorability images a FID score of 104, while the non-constrained images (foils) had a FID score of 88. Based on these minimal differences, it is highly unlikely that differences in image quality are affecting the memorability of our images. Code for this model can be found at https://github.com/ckyleda/MEMGAN, and the VMS training datasets can be found at https://www.cs.york.ac.uk/vischema/.

### Human memory experiment

**Figure 11:**
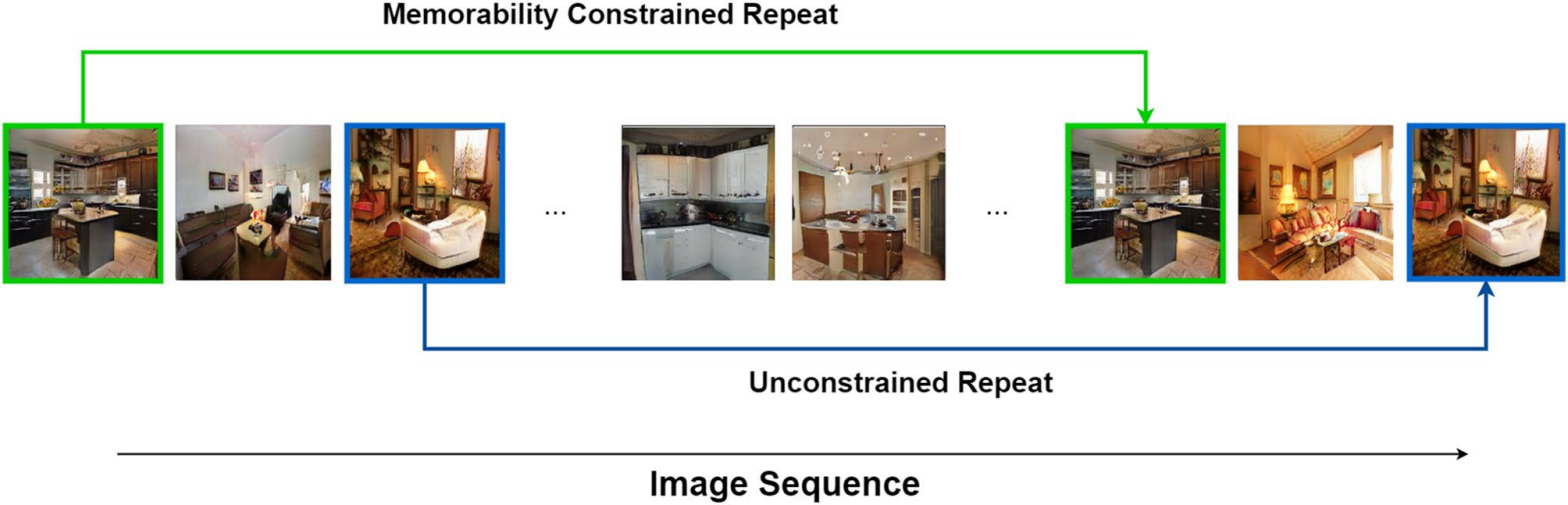
Memorability experiment structure.

With our stimulus set of generated images, we conduct a human recognition memory experiment with 119 participants. Each participant saw one of ten unique sequences of 150 images, with 40 targets (i.e. repeats of once presented images in the sequence) per sequence. Both foils and target images could be repeated. Each image was shown on-screen for 3 s. Where an image was repeated, we ensured a minimum of at least 30 images between first showing and repeat (see Fig. 11). Each sequence was viewed on average by 12 different participants. Participants were asked to indicate by pressing a button when an image they were viewing was a repeat. If they correctly indicated a repeat it was considered a hit and if not, then a miss. When participants indicated that they recognised a repeat, they were asked to annotate the regions in the image they believe caused them to remember the image. This allowed us to gather two-dimensional memorability maps for our generated images. Each sequence took approximately 9 min on average for a participant to complete. We then analyse these results through several statistical tests, primarily a one-way independent Analysis of variance (ANOVA). We also employ Mann–Whitney U tests to verify that our effect occurs in the intended direction, and Kruskal–Wallis and Wilcoxon signed-rank tests to verify that our results hold if distribution assumptions are relaxed. Critically, no single participant was shown both the high-memorability and the low-memorability image of a given pair in the same image stream. This prevented recognition of images by previously viewing the same image with a different memorability value, rather than remembering a repeat of the target. Participants were paid at a rate of $7.02 per h using the crowdsourcing platform Prolific, and prescreened such that all participants were between 18 and 65 years of age and fluent in English. This experiment was approved by the Departmental Ethics Committee of the Dept. of Psychology, University of York, UK, and follows relevant guidelines given by that committee. Informed consent was given by participants, and they were free to withdraw at any time.

### Evaluating scene recognition differences

To determine whether there was any difference in recognizability between high and low memorability images that has arisen due to differences in their structure, we employ a deep neural network. We select a ResNet152 model^24^, which has been pretrained on the Places365^25^ dataset, such that it can classify images into one of 365 different scenes. If the network predicts the image is a kitchen (or kitchen related) we record this as a successful recognition of the scene. As the network predictions can be specific to the *type* of kitchen (for example, the network is capable of differentiating a ‘galley’ style kitchen from a ‘wet-bar’) we select a set of categories that closely relate to kitchens; and assume any prediction in this set is a correct recognition that the image shown is a kitchen. This subset consists of: ‘kitchen’, ‘wet_bar’, ‘galley’, ‘restaurant_kitchen’, and ‘sushi_bar’. We then accumulate predictions by running all images from both the low memorability category and high memorability category through this network, and record the predictions. We find that the low memorability category has a recognition rate (correct predictions) of 95%, and for high memorability, a recognition rate of 96%.
